# A novel design, analysis and 3D printing of Ti-6Al-4V alloy bio-inspired porous femoral stem

**DOI:** 10.1007/s10856-020-06420-7

**Published:** 2020-08-20

**Authors:** Hassan Mehboob, Faris Tarlochan, Ali Mehboob, Seung-Hwan Chang, S. Ramesh, Wan Sharuzi Wan Harun, Kumaran Kadirgama

**Affiliations:** 1 Department of Engineering Management, College of Engineering, Prince Sultan University, Riyadh 11586, Saudi Arabia; 2 Department of Mechanical and Industrial Engineering, College of Engineering, Qatar University, Doha, Qatar; 3 School of Mechanical Engineering, Chung-Ang University, 221, Heukseok-Dong, Dongjak-Gu, Seoul, 156-756 Republic of Korea; 4 Center of Advanced Manufacturing and Material Processing, Department of Mechanical Engineering, Faculty of Engineering, University of Malaya, 50603 Kuala Lumpur, Malaysia; 5 Faculty of Mechanical & Automotive Engineering Technology, Universiti Malaysia Pahang, Gambang, Malaysia

## Abstract

The current study is proposing a design envelope for porous Ti-6Al-4V alloy femoral stems to survive under fatigue loads. Numerical computational analysis of these stems with a body-centered-cube (BCC) structure is conducted in ABAQUS. Femoral stems without shell and with various outer dense shell thicknesses (0.5, 1.0, 1.5, and 2 mm) and inner cores (porosities of 90, 77, 63, 47, 30, and 18%) are analyzed. A design space (envelope) is derived by using stem stiffnesses close to that of the femur bone, maximum fatigue stresses of 0.3σ_ys_ in the porous part, and endurance limits of the dense part of the stems. The Soderberg approach is successfully employed to compute the factor of safety *N*_*f*_ > 1.1. Fully porous stems without dense shells are concluded to fail under fatigue load. It is thus safe to use the porous stems with a shell thickness of 1.5 and 2 mm for all porosities (18–90%), 1 mm shell with 18 and 30% porosities, and 0.5 mm shell with 18% porosity. The reduction in stress shielding was achieved by 28%. Porous stems incorporated BCC structures with dense shells and beads were successfully printed.

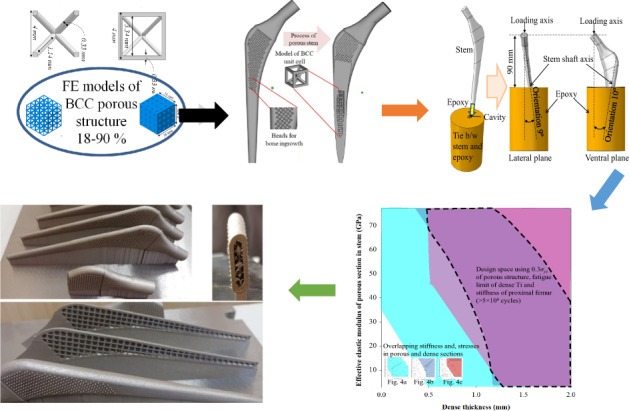

## Introduction

Cementless femoral stems are traditionally used for total hip replacement (THR). The stems are inserted proximally in the medullary canal of the femur and firmly attached to the bone to transfer the physiological load to the bone during daily activities. Ti-6Al-4V alloy (Ti) is proven as an excellent biomaterial [1–4], that exhibits an elastic modulus of 114 GPa, which is ~6–7 times stiffer than the femur. This higher stiffnesses of cementless dense Ti-6Al-4V alloy stems cause several complications such as poor bone ingrowth, stress shielding, risk of bone fracture, and even revision of surgery. Finite element models are used successfully to investigate the biomechanical behavior of tissues and implants [5–15]. Many published works have used finite element models of implants made of composites and porous biomaterials to overcome the aforementioned complications [16–20]. The aim of reducing the stiffness of the femoral stems was achieved using new materials and designs by incorporating different geometrical profiles, porous biomaterials, and functionally graded materials [21–23]. Recently, few studies have introduced new designs of porous and semi-porous femoral stems to reduce the stiffness of the stems [1, 3, 24, 25]. These porous designs are constructed specifically to reduce the stiffness and carry the bodyweight but not for bone ingrowth. Currently, for bone ingrowth, a porous coating (much smaller pores than load carrying porous structure) is fabricated on the proximal side of the femoral stem. Low stiffness porous stems showed lower stress shielding and bone resorption as compared to the dense stems [26]. Many different types of cellular structures were used to obtain the mechanical properties of cellular microstructures. Diamond and body-centered-cubic (BCC) cellular structures provide excellent mechanical properties in terms of compression, bending, and torsional loads [23, 27]; thus, the diamond and BCC types were further used to generate and test porous and semi-porous femoral stems. Additionally, few studies performed static flexural tests and compared the stiffness of the stems and femur [1, 28]. The stresses in the porous and dense Ti-6Al-4V alloy were compared with the yield strengths of porous cellular microstructures, and the stiffnesses of the stems were compared with those of the femur. Several designs of porous and semi-porous stems were suggested after performing static tests although there is a paucity of studies that focus on the fatigue limit of porous stems in a manner that significantly differs from that of dense Ti-6Al-4V alloy. Several studies [22–27] have shown that porous cellular microstructures have fatigue strength fraction to that of the effective yield strength (~0.3σ_ys_) of the cellular microstructure. Hence, reducing stiffness through porous structures is a desired feature, however, the challenge is to ensure the design has a good fatigue limit (>5 × 10^6^ cycles) [29].

**Fig. 1:**
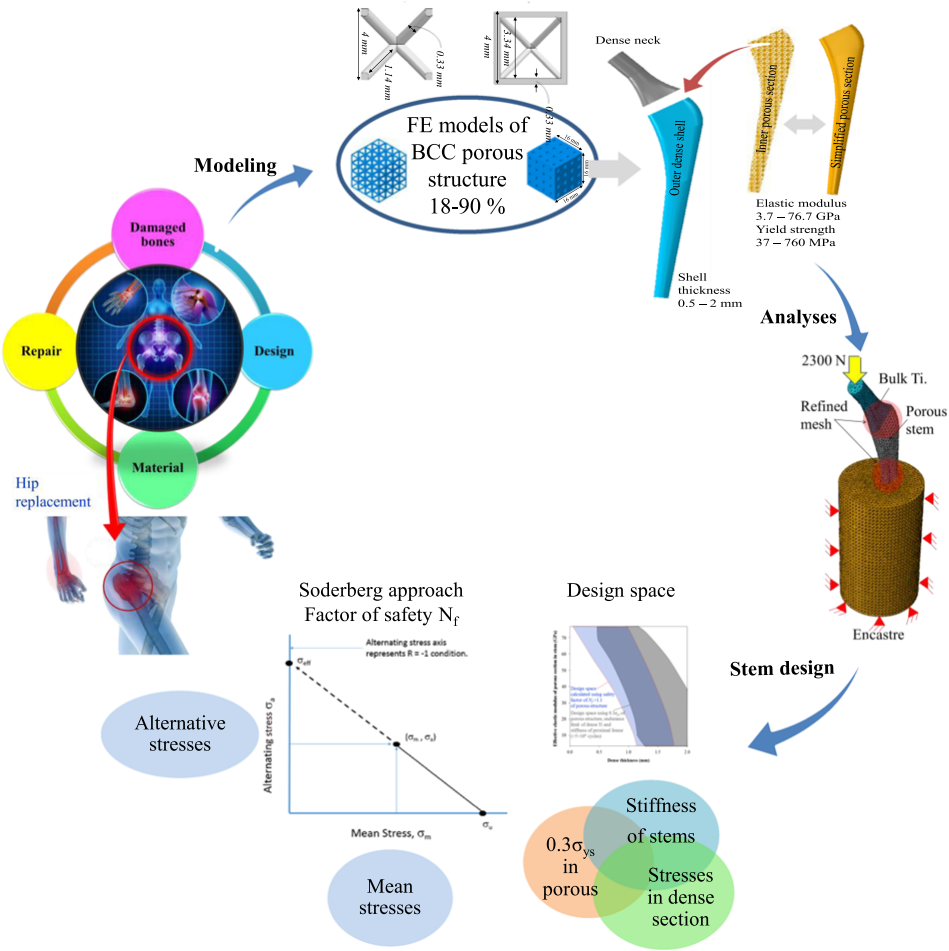
Schematic delineating the pathway to determine the design of porous stems

The objective of this work is to derive a design envelope for cementless Ti-6Al-4V alloy porous stems (see Fig. 1) to reduce the stiffness to match that of a bone and manifest the fatigue limit corresponding to >5 × 10^6^ cycles simultaneously. 3D finite element models of BCC porous structures were constructed and simulated to investigate the mechanical properties. The BCC porous structures with porosities of 18–90% were simulated under compression to yield the effective elastic moduli and the effective yield strengths. The mechanical properties obtained from simulated compression tests of BCC porous structures were used to model solid stems which are named as effective porous stems. 3D finite element models of stems were constructed with various outer dense shell thicknesses and inner sections with different elastic moduli. The ISO 7206-4 [30] standard was used while developing the numerical models of the stems. The proposed enveloped was mapped using the stiffnesses of the stems and stresses in porous and dense sections of the stems that exhibited a fatigue limit (>5 × 10^6^ cycles). Soderberg approach was also used to compute the factor of safety [31] under fatigue loads.

## Materials and methods

### Finite element models of BCC porous microstructures

**Fig. 2:**
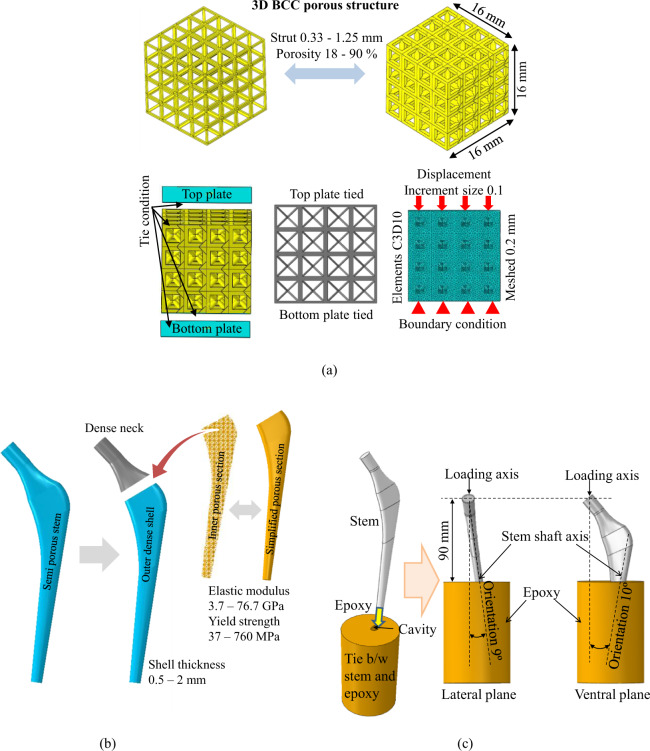
Finite element models; **a** details of porosity, loading and boundary conditions of BCC porous structures, **b** construction of 3D finite element model of the femoral stem and **c** testing method of femoral stem according to ISO 7206-4 standard

In the present study, 3D FE models of the BCC microstructures were constructed using computer-aided design (CAD) software (SolidWorks 15, USA) and simulated in ABAQUS software (version 6.17; ABAQUS Inc., Providence, RI, USA). Porosities of 18–90% were designed using a strut thickness of 0.33–1.25 mm as listed in Table 1. A sensitivity analysis of effective mechanical properties was performed with different numbers of BCC cubes (1, 2, 3 and 4 cubes) with dimensions of 4 × 4 × 4 mm^3^ each cube that resulted the BCC structures with dimensions of 4 × 4 × 4, 8 × 8 × 8, 12 × 12 × 12, 16 × 16 × 16 mm^3^ in our previous study [23]. This analysis revealed that BCC structure with dimensions of 16 × 16 × 16 mm^3^ (four cubes in each direction) did not change the effective mechanical properties further increasing the number of cubes. Therefore, BCC structure with dimensions of 16 × 16 × 16 mm^3^ was modeled that mimics the macro structural behavior as shown in Fig. 2a. The elastic–plastic material properties of dense Ti-6Al-4V alloy (elastic modulus 114 GPa) were used to assign the BCC structures [32]. A mesh sensitivity analysis was performed with element sizes of 0.1, 0.2, 0.3, 0.4 mm and a mesh size of 0.2 mm converged the results accuracy in our previous study [23]. Tetrahedral elements (C3D10) with a mesh size of 0.2 mm was used in this study. A bottom and a top rigid plate are tied to the BCC structure to apply the boundary conditions as shown in Fig. 2a. The bottom plate was employed with the encastre boundary condition, and the top plate was subjected to the displacement boundary condition to achieve the yield point. The stress-strain curves, effective elastic moduli and effective yield strengths of porosities in the range of 90–18% were calculated from the compression tests. The fatigue limit of cellular porous structures was reported as ~20–30% of the yield strength [33–35]. To predict the fatigue limit of BCC porous cellular structures, the stresses were obtained from 20–100% of the yield strength. The procedure was adopted for all the porosities in 18–90% of BCC porous structures. The finite element models of BCC porous structures were simulated and results were compared with experimental data [27], and the results of effective elastic modulus and yield strength were validated in our previous study [23].

**Table 1:** Properties of BCC Ti-6Al-4V porous microstructure

Strut thickness (mm)	Porosity (%)	Effective elastic modulus (GPa)	Effective yield strength, σ_ys_ (MPa)	0.3σ_ys_ (MPa)
0.33	90	3.8	37	11
0.51	77	9.1	89	27
0.69	63	18.3	177	53
0.87	47	31.5	305	91
1.08	30	53.8	529	159
1.25	18	76.7	760	228

### Validation of finite element models of stems

3D models of porous and effective porous stem were constructed in computer-aided design (CAD) software (SolidWorks 15, USA) and Materialise Magics® (version 21.1; Materialise NV, Leuven, Belgium), and simulated in ABAQUS software (version 6.17; ABAQUS Inc., Providence, RI, USA). The aim of constructing these models was to validate our finite element models. Therefore, a porous stem with a porosity of 63% is simulated and the elastic-plastic mechanical properties of bulk Ti-6Al-4V alloy was assigned to a porous section as shown in Fig. 2b. Another 3D model of effective porous stem was simulated and effective mechanical properties of 63% porous structure is assigned to a solid stem. The hypothesis to model an effective porous stem instead of a porous stem was to reduce modeling and computational time. To validate the finite element models, force-displacement data of porous stem and effective porous stem were plotted and compared.

### Finite element models and design of experiments

3D finite element models of the effective porous stem stems were constructed computer-aided design (CAD) software (SolidWorks 15, USA). The effective elastic moduli and effective yield strengths were obtained from simulated compression tests of BCC porous structures for all porosities 18–90%. The 3D finite element models of stems were simulated in ABAQUS software (version 6.17; ABAQUS Inc., Providence, RI, USA). The porous stems were modeled with and without outer dense shells made of dense Ti-6Al-4V alloy and an inner core was modeled with different effective material properties of porosities in the range of 18–90%, as shown in Fig. 2b. The stem neck was modeled as a fully dense Ti-6Al-4V alloy throughout this study. The thickness values of the outer dense shell correspond to 0.5, 1.0, 1.5, and 2 mm, and the porosity of the porous section was 18–90%. The stress–strain data of BCC porous microstructures were used for the inner sections of the stems. A design of experiments (DOE) technique was used to design the simulation runs as listed in Table 2. A combination of five thicknesses of outer dense shells and six porous sections resulted in 30 computer simulations for porous stems. A total of 30 simulations were designed for the computer simulation.

**Table 2:** Design of experiments (DOE) of computer simulations for porous stems

Computer simulations	Dense shell thickness (mm)	Porosity of porous section (%)	Stiffness of stems (N/mm)	Maximum stresses in dense shell (MPa)	Maximum stresses in porous section (MPa)
1	–	90	134	–	104
2	–	77	298	–	136
3	–	63	542	–	217
4	–	47	844	–	312
5	–	30	1284	–	296
6	–	18	1671	–	289
7	0.5	90	900	969	31
8	0.5	77	999	848	64
9	0.5	63	1141	709	106
10	0.5	47	1321	583	147
11	0.5	30	1593	452	190
12	0.5	18	1845	368	217
13	1.0	90	1340	578	15
14	1.0	77	1412	540	35
15	1.0	63	1508	493	65
16	1.0	47	1626	445	101
17	1.0	30	1803	383	148
18	1.0	18	1967	337	184
19	1.5	90	1639	445	10
20	1.5	77	1691	427	25
21	1.5	63	1757	403	47
22	1.5	47	1835	378	76
23	1.5	30	1949	345	117
24	1.5	18	2055	318	152
25	2.0	90	1845	379	8
26	2.0	77	1883	368	19
27	2.0	63	1928	355	37
28	2.0	47	1978	340	62
29	2.0	30	2050	321	99
30	2.0	18	2115	305	133

#### Finite element model for fatigue tests

The stems were designed to withstand walking loads (cyclic), and thus the ISO 7206-4 standard was followed to test the fatigue behavior of the stems (Fig. 2c). Finite element code ABAQUS (version 6.17; ABAQUS Inc., Providence, RI, USA) was used to simulate the stems throughout this study. As per ISO 7206, the FE models of the stems were inserted into an epoxy cylinder. The stems were orientated 10° in the ventral plane and 9° in the lateral plane while maintaining a distance of 80 mm between the surface of the epoxy and the head of the stem as shown in Fig. 2c.

#### Materials, loads, and boundary conditions

The effective elastic moduli and effective yield strengths of BCC porous structures with porosities of 18–90% were assigned to the porous section of the stems. Elastic moduli of 114 GPa (elastic–plastic properties of bulk Ti-6Al-4V alloy) and 3.7 GPa (linear elastic) were used for the outer dense shell and epoxy, respectively. In this study, the body-weight was assumed as 800 N with contact forces on the hip joint as 250–300% of the body-weight [36, 37]. The contact force at the hip corresponds to a load of 2.3 kN applied on the head of the stem following the standard. A stress ratio of R = 0.1 was used in the fatigue test (minimum contact force of 230 N) and C3D10 tetrahedral elements were assigned to the stem and epoxy (mesh size 1 mm).

### Fatigue analysis of porous stems

Finite element analyses (FEA) was performed to estimate the axial stiffnesses of the stems. The design envelope was developed by considering (a) the effective femur bone stiffness of the stem, (b) 0.3σ_ys_ stresses in the porous sections with regards to the fatigue limit of porous structures [33–35, 38] and (c) 500-MPa outer dense shell (fatigue strength of 3D printed Ti-6Al-4V alloy is 500 MPa) [39]. Overlapping the three spaces determined the fatigue design envelope. Soderberg theory was used to calculate the factor of safety [31]. This theory uses the mean (*σ*_*m*_) and alternating (*σ*_*a*_) stresses in computing the factor of safety (Eqs. (1–4)). Minimum (*σ*_min._) and maximum stresses (*σ*_max._) in the porous section and outer dense shell were calculated using the minimum (230 N) and maximum (2300 N):

1 $$\sigma _m = \frac{{\left( {\sigma _{\max .} + \sigma _{\min .}} \right)}}{2}$$

2 $$\sigma _a = \frac{{\left( {\sigma _{\max .} - \sigma _{\min .}} \right)}}{2}$$

The Soderberg equation is used to calculate the factor of safety in Eq. (3) as follows:

3 $$\left( {\frac{{\sigma _a}}{{S_e}}} \right) + \left( {\frac{{\sigma _m}}{{S_{ys}}}} \right) = \frac{1}{N}$$

The fatigue factor of safety *N*_*f*_ is as follows:

4 $$N_f = \frac{1}{{\frac{{\sigma _a}}{{S_e}} + \frac{{\sigma _m}}{{S_{ys}}}}}$$

**Fig. 3:**
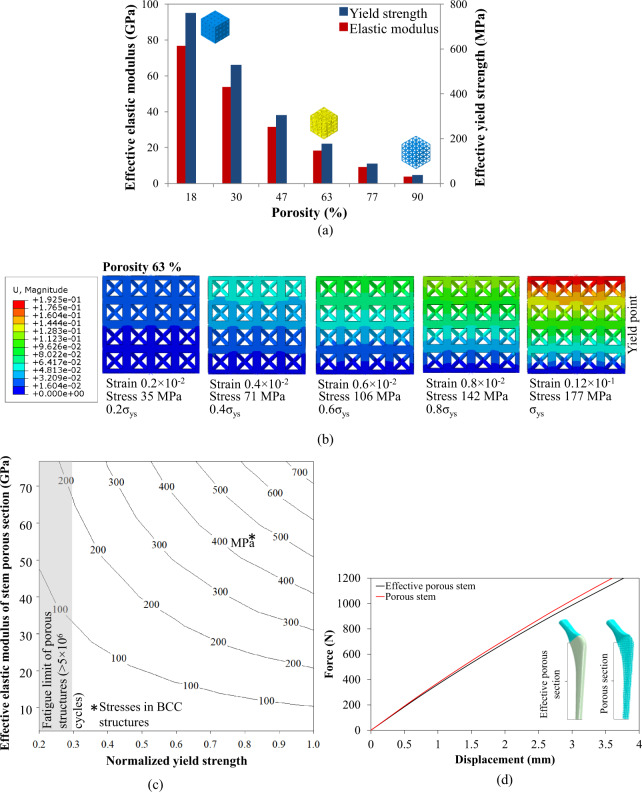
Results of finite element models; **a** effective mechanical properties versus porosity, **b** different stresses and strains at different displacements, **c** calculation of 0.3σ_ys_ as endurance limit and **d** validation results of solid and porous stems

Whereas, *S*_*e*_, *S*_*ys*_, and *N* denote the endurance limit of the material, yield strength of the material, and factor of safety, respectively. For the dense outer shell, endurance limit and yield strength of 3D printed direct metal laser sintering (DMLS) Ti-6Al-4V alloy correspond to 500 and 1180 MPa [39], respectively. The endurance limit and yield strength envelope for the porous Ti-6Al-4V alloy is shown in Fig. 3. This is based on the fact that any combination of mean and alternating stress that lies on or below the Soderberg line is considered safe for design [31].

### Prototype printing of stems

This study aimed to analyze the design of dense and porous Ti-6Al-4V alloy stems and print using the DMLS technique to reduce the stiffness and provide adequate fatigue life. Therefore, porotypes of dense and porous stems were printed using the EOS M280 printer. The original EOS parameters of performance-based were selected to print the stems. The layer thickness was set to 30 µm and laser power was set to 200 watts. Default scanning speed and laser parameters were set for the printing of Ti-6Al-4V alloy. Argon gas was supplied to the printing chamber throughout the printing session.

## Results

Different porosities of BCC microstructures were simulated under the uniaxial compression test to compute the effective mechanical properties. The effective elastic moduli and effective yield strengths of porosities in the range of 18–90% were 76.7–3.8 GPa and 760–37 MPa, respectively, as shown in Fig. 3a and listed in Table 1. The effective mechanical properties of BCC porous microstructures were used for modeling the stems. To predict the fatigue limit of BCC porous cellular structures (e.g., the porosity of 63%), stresses were obtained from 20–100% of the yield strength (37–177 MPa) with strains in the range of 0.002–0.012 mm/mm as shown in Fig. 3b. This process was adopted for all the porosities (18–90%), and normalized yield strength and effective elastic moduli were plotted as shown in Fig. 3c. The gray shaded area in Fig. 3c denotes 30% yield strength (11–235 MPa) of BCC porous structures with porosities of 18–90%, which is considered as the safe region of fatigue limit (>5 × 10^6^ cycles) for orthopedic implants [34]. The values of stresses under this region were used to design the porous sections of the stems. All the stems were oriented according to the ISO 7206-4 standard to compute the stiffness, fatigue limit and design space for porous stems. Finite element models were validated using force-displacement data of porous stem and effective porous stem which were plotted and compared in Fig. 3d.

### Design of stems by considering fatigue limit

#### Mechanical properties of BCC porous structures

To investigate the fatigue life of the porous structures, the stresses and the strains were obtained at different load points below the yield point. These points included the data of 30% stresses of the yield strength for a specific porosity. As the porosity was changed, the yield point and 30% stresses of yield strength were changed. Figure 3b shows the stresses, strains and yield point of a 63% BCC porous structure. For 63% BCC porous structure, the yield point was determined at 177 MPa with a strain value of 0.012 mm/mm. However, 30% of the yield strength is 35–53 MPa which was considered to be safe under fatigue loads for this specific porosity. Therefore, this procedure was adopted for the porosities 18–90% yielding the elastic moduli of 3.8–76.7 GPa were plotted in Fig. 3c. The gray shaded area in Fig. 3c shows 30% of yield strength of porosities 18–90%.

#### Model validation of porous and effective porous stems

Porous and effective porous stems were validated as shown in Fig. 3d. When a force of 1200 N was applied to the stems, very close displacements of 3.7 and 3.8 mm are observed for porous and effective porous stems, respectively. This result is the evidence that an effective porous stem can be successfully used for finite element simulations instead of a real porous stem after carefully obtaining the elastic-plastic mechanical properties of porous structures of different porosities.

#### Stiffness of porous stems

**Fig. 4:**
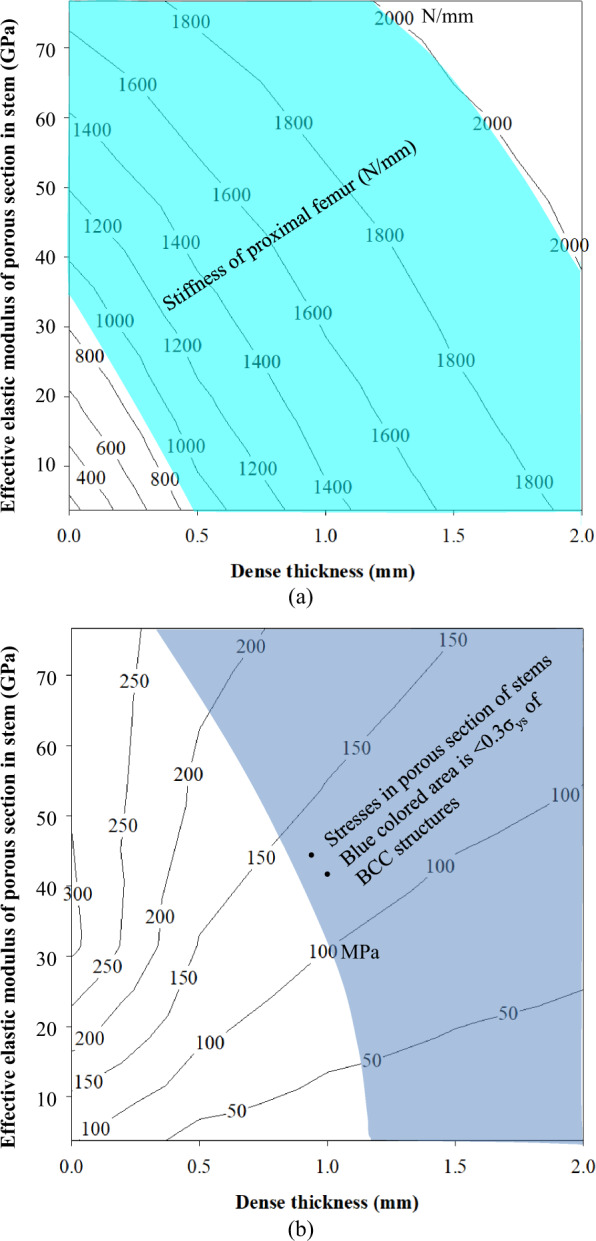
Results of stiffness and stresses in porous stems; **a** stiffness of the stem matches to the bone represented cyan shaded area; **b** 0.3σ_ys_ stresses in porous section of stems represented blue shaded area (Color figure online)

As shown in Fig. 4a, stiffnesses of the porous stems are plotted via the FEA results. The cyan shaded area in Fig. 4a denotes the stiffness values of the femur bone (900–2000 N/mm) [40, 41]. Stiffness of the stem is a combination of outer dense shell and effective elastic moduli of porous sections. Fully porous stems without an outer dense shell with different effective elastic moduli (3.8–76.7 GPa) yield a stiffness of 200–1650 N/mm as shown in Fig. 4a. Similarly, the stiffness values are determined for dense shells with thicknesses of 0.5, 1.0, 1.5, and 2 mm. Further increasing the thickness of the dense shell outside the effective porous stem section, increased the stiffness of the stems. Similarly, increasing the effective elastic modulus of the stem showed higher stiffness of the stem. 0.5 thick dense shell gave the stiffness 900–1820 N/mm for effective elastic moduli of 3.8–76.7 GPa. Increasing the thickness of the dense shell to 1 mm with effective elastic moduli of 3.8–76.7 GPa gave the stiffness of 1380–1880 N/mm. Similarly, the dense shell thicknesses of 1.5 and 2 mm with effective elastic moduli of 3.8–76.7 GPa showed the stiffness of 1640–2040 and 1830–2180 N/mm, respectively.

#### Stresses in effective porous section

As shown in Fig. 4b, the maximum stresses generated in the fully porous stems with elastic moduli of 3.8–76.7 GPa correspond to 104–289 MPa. Fully porous stems with elastic moduli of 3.8, 9.1, 18.3, and 31.5 GPa indicate higher stresses (104–312 MPa) than their yield strengths (37–305 MPa). These stems were yielded and could not even withstand static loads. Increases in the elastic moduli from 53.8 to 76.7 GPa (30 and 18%) of the fully porous stems showed stresses 289 and 296 MPa, respectively, which are lower than the effective yield strengths (529 and 760 MPa). Although the stresses in 53.8 and 76.7 GPa effective porous stems are still higher than the allowable fatigue limit (in blue shaded area) of 0.3σ_ys_ as shown in Fig. 4b and Table 1 which are 159 and 228 MPa, respectively. Other studies only considered the yield strength to design the stem [1, 32, 42] that may fail under the fatigue loads. Thus, 0.2–0.3σ_ys_ exhibits the desired limit of porous structures under fatigue loads [33, 34]. Thus, the design of the stem indicates that the elastic modulus of the fully porous stem is not sustained with the desired fatigue limit. However, introducing an outer dense shell thickness of 0.5 mm with an effective elastic modulus of 76.7 GPa stem exhibits stresses 225 MPa lower than 0.3σ_ys_ which are 228 MPa as shown in Fig. 4b and Table 1. Similarly, a 1-mm dense shell with effective elastic moduli of 53.8 and 76.7 GPa stems showed the stresses 155 and 170 MPa, respectively, which are lower than 0.3σ_ys_ 159 and 228 MPa, respectively. However, effective porous sections of all effective elastic moduli stems with dense shells of 1.5- and 2-mm thickness shows stresses less than 0.3σ_ys_ as shown in Fig. 4b and Table 1.

#### Stresses in outer dense shell

**Fig. 5:**
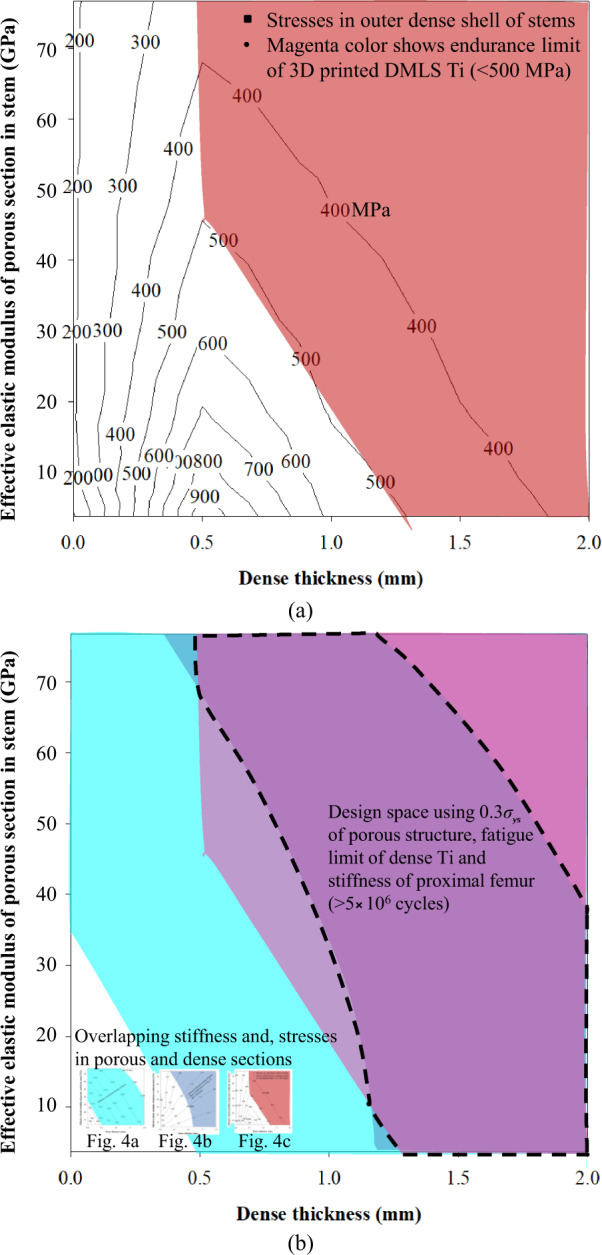
Results of stresses in dense section and design space of porous stems; **a** stresses in dense outer shell shown in magenta color to be safe under fatigue load, **b** a design space determined using stiffness, stresses in porous section and stresses in a dense section (Color figure online)

To estimate the fatigue limit of the outer dense shell, the maximum von Mises stresses are obtained from simulated results as shown in Fig. 5a. The magenta color area denotes the safe stresses (max. 500 MPa) in the dense outer shells of stems. The plot also provides the freedom to select the shell thickness. Additionally, 0.5- and 1-mm thick dense shells with porous sections of elastic moduli 47–76.7 and 20–76.7 GPa, respectively, falls within magenta color area. However, stems with dense shell thickness values corresponding to 1.5- and 2-mm with any elastic moduli of the porous section falls within the magenta color area as shown in Fig. 5a. These results show how the thickness of the dense shell is selected while designing the stems to be safe under fatigue loads. The plots of shaded areas of stiffness (Fig. 4a), stresses in porous section (Fig. 4b), and stresses in outer dense (Fig. 5a) shell are overlapped as shown in Fig. 5b. The overlapping of these plots will determine the space that allows freedom to design the stems with different stiffness and porosities without failure under the fatigue loads as shown in Fig. 5b.

### Calculation of the safety factor using the Soderberg approach

**Fig. 6:**
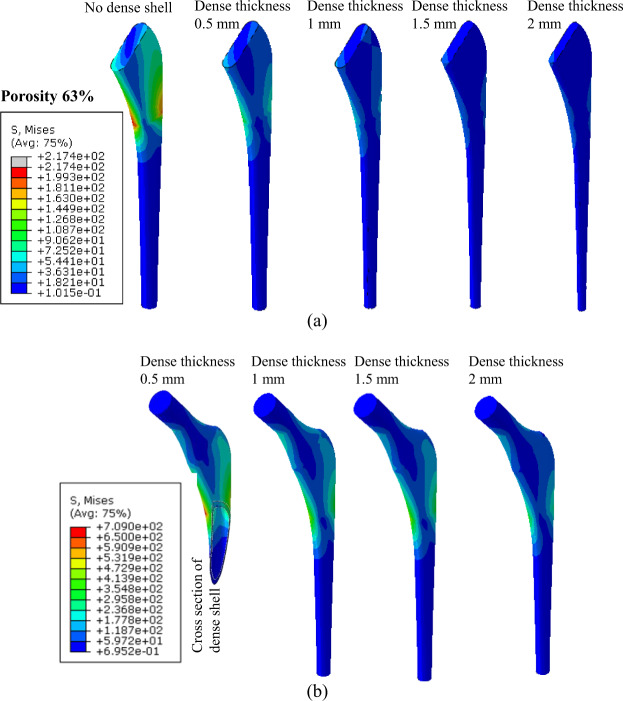
Von Mises stresses (MPa) in femoral stem with a porosity of 63%; **a** stresses in the porous section of stem with different outer shell thickness, **b** stresses in outer dense shell with different thickness

**Fig. 7:**
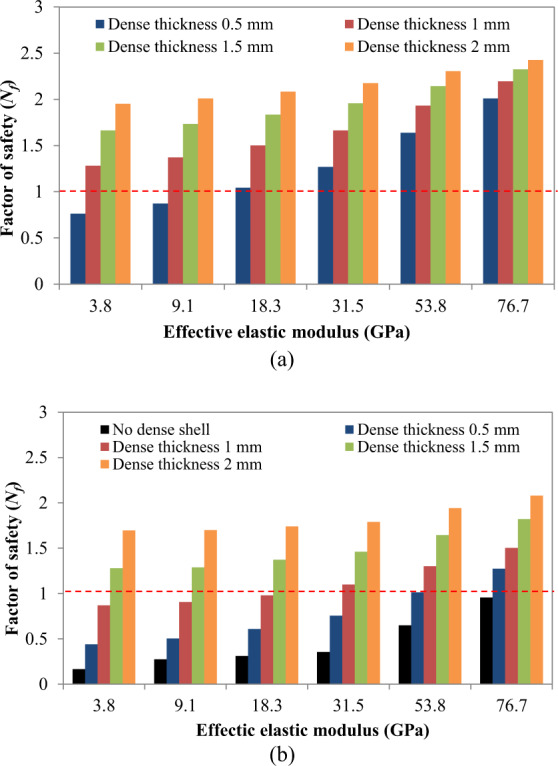
Utilization of Soderberg approach to calculate the factor of safety; **a** factor of safety calculated for outer dense shell with different porous stems, **b** factor of safety calculated for porous section with different porosities

**Fig. 8:**
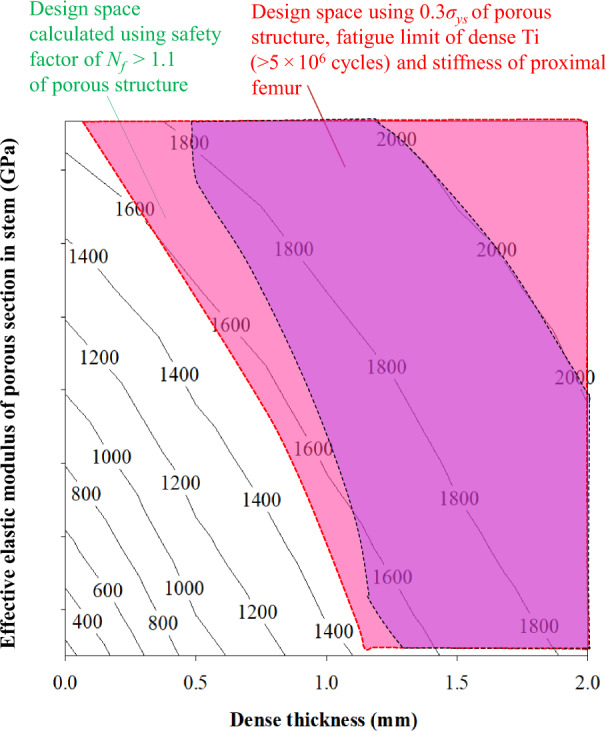
Design spaces calculated using the Soderberg approach for the safety factor of *N*_*f*_ > 1.1 and, stiffness, stresses in porous section and stresses in dense sections

**Fig. 9:**
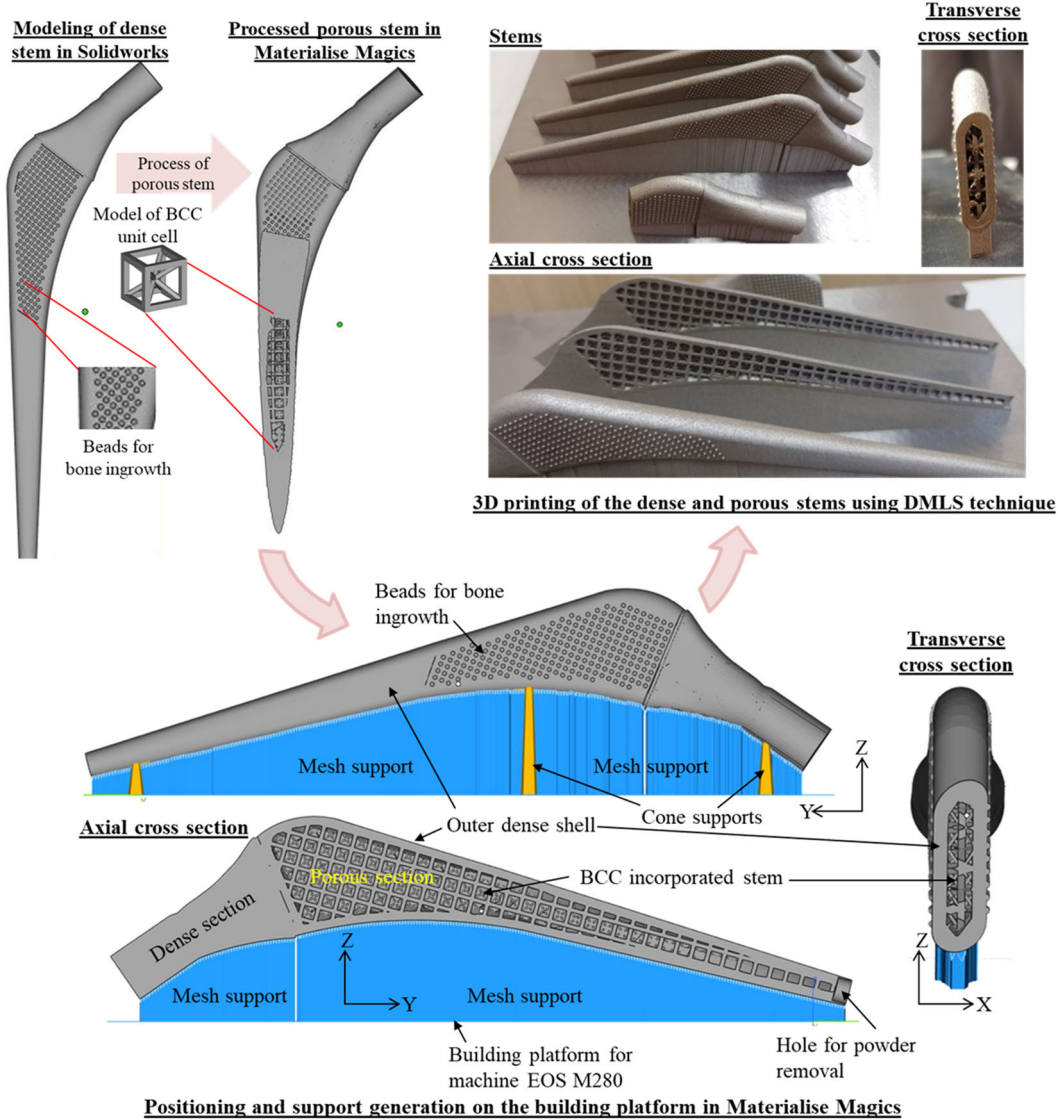
3D printed porous stems with outer beads using the DMLS technique

Stresses in all sections of the stem are computed for all effective elastic moduli as shown in Fig. 6a, b, respectively. The stresses were decreased in the porous sections of the stems when the thickness of the dense shell was increased. Figure 7 depicts the factor of safety for various sections of stems with different effective elastic moduli. As shown in Fig. 7b, all porous sections with outer dense shells with thickness values of 1.5 and 2 mm survive under the fatigue load. The results obtained from the first method for the prediction of fatigue limit and the second method using the Soderberg equation were plotted in Fig. 8. This plot gives the range of the stems to be fabricated with different stiffnesses according to the patient’s need. The prototype of porous stems with outer beads is printed using the DMLS technique as shown in Fig. 9. The outer beads will facilitate the porous space for the bone ingrowth and inside the porous structure of the stem will provide reduced stiffness with enough fatigue life. The testing of these stems is part of our future study.

### Stress shielding of dense and porous stems from design space

Dense stems and porous stems with outer dense sleeve (from design space in Fig. 8) were selected to investigate their performance in bone. Different porous stems with different outer sleeves were selected and inserted into the bone as listed in Table 3. The stiffness of the stems was calculated from the contour plot of the stiffness relationship between dense sleeve thickness and inner. The walking loads were applied to the stem and the bone. The most crucial zone of bone resorption is the calcar region (Gruen zone 7) in cancellous bone where the stress shielding affects the most. Which further cause loosening of implants, immature failure and revision of surgery. The results yield that stress shield was achieved from 8 to 28% lesser than dense Ti-6Al-4V alloy stem when different porous stems with different dense shell thicknesses were implanted into the bone as listed results in Table 3. This study aimed to design the stems in a way to reduce the stress shielding up to 30% that is quite close to our achieved results.

**Table 3:** Stress shielding in cancellous bone under walking load

Stem	Stiffness of stem (N/mm)	Stresses in cancellous bone (MPa)	Reduction in stress shielding in cancellous bone (%)
17 GPa (intact bone)	1546	21	–
Dense	2212	9	
1 mm dense sleeve + 31 GPa porous inside	1626	12	28
0.5 mm sleeve + 76 GPa porous	1844	10	8
1 mm sleeve + 76 GPa porous	1967	10	10
1.5 mm sleeve + 4 GPa porous	1639	12	29
1.5 mm sleeve + 54 GPa porous	1949	10	14

## Discussion

### Fatigue life of porous cellular structures

The finite element models of BCC porous structures were validated with the published data [27], and the procedure was explained in our previous study [23]. BCC porous structures with porosities of 18–90% showed the elastic moduli of 3.8–76.7 GPa as shown in Fig. 3a. The elastic moduli and yield strengths of 77–63% porosities were 9.1–18.3 GPa and 89–177 MPa, respectively, which falls within the properties of the material of cortical bone (elastic moduli 8–20 GPa and yield strengths 85–108 MPa) [43, 44]. Among these densities, the most closely matched material properties of 63% BCC porous structure to the bone which presented the elastic modulus and yield strength of the femur of most of the population. However, the bone is living tissue and new cells are developed continuously which does not fail under fatigue loads of human daily activities. Therefore, while designing a porous artificial organ via an additive manufacturing process, fatigue life should be kept in mind. The fatigue life of porous cellular biomaterials is much lower than yield strength [29, 33]. Ahmadi et al. [29] reported that satisfactory fatigue life of porous cellular biomaterials was achieved when they were subjected to 30–40% stresses of yield strength. Figure 3b presented the stresses in BCC porous structures with different porosities at different strain points till the yielding was achieved. Yavari et al. [38] has presented that the porous structures completed 10^6^–10^5^ loading cycles when 30% stress level of the yield strength was applied with a stress ratio of R = 0.1. Figure 3c showed the gray color area which presented the 30% stresses of the yield strength for all the porosities.

### Model validation

The model validation showed reasonable agreement between porous stem and effective porous stem as showed in Fig. 3d. The global behavior of the force-displacement plot of porous and effective porous stems was observed in Fig. 3d. Jette et al. [28] presented a finite element model of effective porous stem and compared the displacement and strains fields in the simulated stem model and printed stem. The correlation between experimental and simulated stem showed strong agreement. Similarly, in this study effective porous stem showed displacement close to the porous stem which is the evidence of validation of our model.

### Fatigue life of porous stems

The porous stems were designed considering the yield strength and elastic modulus of porous cellular materials [1, 2, 28]. The properties of the stems were compared to the cortical and the trabecular bones. In the finite element models, the stresses in porous stems were compared with their yield strength in the literature [28], however, the porous stems fail under fatigue life far below their yield strengths. Similarly, Hazlehurst et al. [30, 45] designed the stems considering their flexural stiffnesses. Effective elastic moduli and effective yield strengths of different porosities were obtained. The flexural stiffness of these porous stems was compared with the stiffness of the bone without considering the fatigue behavior of porous cellular structures. Therefore, in this study, the fatigue life of effective porous stem was predicted using two methods; one method includes 30% stresses of the yield strength of all porosities and the stiffness of the stems matching to the bone, and other method includes the process followed by the Soderberg equation to predict the fatigue limit. Several studies have predicted the fatigue limit using finite element models of the solid stems [31, 46], however, no literature was found on the prediction of fatigue life of finite element models of porous stems. In these studies, Soderberg and Goodman’s theories were utilized to estimate the fatigue life after calculating the maximum and minimum stresses in the solid fully dense stems. The fatigue behavior of bulk material is not as complex as that of porous cellular materials. Therefore, designing the porous stems needs a deep understanding of the fatigue behavior of porous cellular structures. In this study, Soderberg theory was utilized and maximum and minimum stresses were obtained in effective porous stems when cyclic stress was applied and R = 0.1. The factor of safety was calculated and compared with the determined design space in the first method (30% stresses of yield strength). The determined design space of effective porous stems and factor of safety showed the convergence of the results, hence, the porous stems within the design space can be used efficiently. The design space gives the freedom to the surgeons to select the porous stem with different stiffness according to the bone quality of the patient. The design of one stem cannot be utilized for all the patients due to the heterogeneity of the bone. Therefore, different types of stems are commercially available such as short stem and long stems, and modular and non-modular stems, and so on.

The limitation of this study includes the clinical data availability and the response of the bone to the porous stems is not studied yet. Before clinical testing, the 3D printed porous stems (see Fig. 9) need to be tested under the fatigue loads to investigate the fatigue limit influenced by manufacturing defects, and porosity differences between CAD models and printed stems. Moreover, the effect of polishing, heat treatment, surface roughness, topography, and chemistry strongly affects both the osseointegration and the fatigue resistance, which is the part of the ongoing project and future study.

## Conclusions

The goal of this study was to focus on cementless femoral stems in terms of developing a fatigue design space (envelope). Here, FE models of porous stems with various porosities were constructed. To obtain the mechanical properties, the structures were subjected to uniaxial compressive loads. It was found that the modulus and effective yield strengths corresponded to 3.8–76.7 GPa and 37–760 MPa, respectively. Stress–strain data from compression tests were used for 3D finite element models of the stems. ISO 7206-4 standard was used in developing these FE models along with appropriate fatigue theory. The following conclusions were obtained from this study:

1. The mechanical properties of porous structures varied by changing the porosity and it affects the overall performance in terms of fatigue.
2. 0.3σ_ys_ criterion and Soderberg approach was used to design the porous stems under fatigue loads.
3. A design space was developed that allows designers to design femoral stems based on patients requirements in terms of stiffnesses matching those of the bone.
4. The reduction in stress shielding was achieved by up to 28% as compared to dense Ti-6Al-4V alloy stem.
